# Supplementary material for: Decreased Indian Ocean Dipole variability under prolonged greenhouse warming
Source: Nat Commun. 2024 Apr 1;15:2811. doi: 10.1038/s41467-024-47276-7 (PMC10985080; doi:10.1038/s41467-024-47276-7)
Supplement: Supplementary file 1 — Supplementary Info [file 41467_2024_47276_MOESM1_ESM.pdf]

Supplementary Information for

Decreased Indian Ocean Dipole variability under prolonged greenhouse warming

Soong-Ki Kim[1], Hyo-Jin Park[1,2], Soon-Il An[1,2,3*], Chao Liu[1], Wenju Cai[4,5,6,7], Agus Santoso[8,9,10], Jong-Seong Kug[11]

[1]Irreversible Climate Change Research Center, Yonsei University, Seoul, Republic of Korea

[2]Department of Atmospheric Sciences, Yonsei University, Seoul, Republic of Korea

[3]Division of Environmental Science and Engineering, Pohang University of Science and Technology (POSTECH), Pohang, Republic of Korea

[4]Frontiers Science Center for Deep Ocean Multispheres and Earth System/Physical Oceanography Laboratory/Sanya Oceanographic Institution, Ocean University of China, Qingdao, China

[5]Laoshan Laboratory, Qingdao, China

[6]State Key Laboratory of Marine Environmental Science & College of Ocean and Earth Sciences, Xiamen University, Xiamen, China

[7]State Key Laboratory of Loess and Quaternary Geology, Institute of Earth Environment, Chinese Academy of Sciences, Xi'an, China

[8]Centre for Southern Hemisphere Oceans Research (CSHOR), CSIRO, Hobart, Australia

[9]Climate Change Research Centre and Australian Research Council (ARC) Centre of Excellence for Climate Extremes, The University of New South Wales, Sydney, Australia

[10]International CLIVAR Project Office, Ocean University of China, Qingdao, China

[11]School of Earth and Environmental Sciences, Seoul National University, Seoul, Republic of Korea

*Corresponding author: Soon-Il An (sian@yonsei.ac.kr)

This PDF file includes:

Supplementary Tables 1 to 3

Supplementary Figures 1 to 30

Supplementary Discussions 1 to 3

Supplementary Table 1. Model and experiment list of the LongRunMIP ensemble.

Model name	Experiment	Total simulation length (years)
CCSM3	control	1530
CCSM3	abrupt2x	3000
CCSM3	abrupt4x	2120
CCSM3	abrupt8x	1450
CCSM3II	control	3805
CCSM3II	abrupt700ppm	3701
CCSM3II	abrupt1400ppm	3132
CESM1.0.4	control	1000
CESM1.0.4	abrupt2x	2500
CESM1.0.4	abrupt4x	5900
CESM1.0.4	abrupt8x	5100
GFDL-CM3	control	5200
GFDL-CM3	1pct2x	5200
GFDL-ESM2M	control	1340
GFDL-ESM2M	1pct2x	4500
GISS-E2-R	control	5525
GISS-E2-R	1pct4x	5001
GISS-E2-R	abrupt4x	5001
HadCM3L	control	1000
HadCM3L	abrupt2x	1000
HadCM3L	abrupt4x	1000
HadCM3L	abrupt8x	1000
IPSL-CM5A-LR	control	1000
IPSL-CM5A-LR	abrupt4x	1000
MIROC3.2	control	681
MIROC3.2	1pct2x	2003
MIROC3.2	1pct4x	2002

Supplementary Table 2. Model list of the CMIP6 piControl experiment.

Model name	Ensemble	Total simulation length (years)
ACCESS-CM2	r1i1p1f1	500
ACCESS-ESM1-5	r1i1p1f1	900
BCC-CSM2-MR	r1i1p1f1	600
CESM2	r1i1p1f1	1200
CESM2-WACCM	r1i1p1f1	499
CMCC-CM2-SR5	r1i1p1f1	500
CMCC-ESM2	r1i1p1f1	500
CNRM-CM6-1	r1i1p1f2	500
CNRM-CM6-1-HR	r1i1p1f2	300
CNRM-ESM2-1	r1i1p1f2	500
CanESM5	r1i1p1f1	1000
CanESM5-CanOE	r1i1p2f1	501
E3SM-1-1	r1i1p1f1	165
EC-Earth3	r1i1p1f1	501
EC-Earth3-CC	r1i1p1f1	505
EC-Earth3-Veg	r1i1p1f1	500
EC-Earth3-Veg-LR	r1i1p1f1	501
FGOALS-f3-L	r1i1p1f1	500
FGOALS-g3	r1i1p1f1	700
FIO-ESM-2-0	r1i1p1f1	475
GFDL-CM4	r1i1p1f1	500
GFDL-ESM4	r1i1p1f1	500
GISS-E2-1-G	r1i1p1f2	345
HadGEM3-GC31-LL	r1i1p1f1	500
INM-CM4-8	r1i1p1f1	531
INM-CM5-0	r1i1p1f1	1201
IPSL-CM6A-LR	r1i1p1f1	1200
KIOST-ESM	r1i1p1f1	150
MIROC-ES2L	r1i1p1f2	500
MIROC6	r1i1p1f1	800
MPI-ESM1-2-LR	r1i1p1f1	1000
MRI-ESM2-0	r1i1p1f1	701
NESM3	r1i1p1f1	500
NorESM2-LM	r1i1p1f1	501
NorESM2-MM	r1i1p1f1	500
UKESM1-0-LL	r1i1p1f2	1100

Supplementary Table 3. LongRunMIP model evaluation summary. The low performance model, which is excluded from the analysis in the main paper, is marked as gray shading.

Type	Product	Peak DMI Standard Deviation Season	DMI Skewness Sign	DMI Regression Pattern (Peak Positions)	Model Performance Level (Score)
Observation (1900-2022)	ERSSTv5	October	Positive	Dipole (Sumatra-Java coast / East African coast)	-
	COBE	October	Positive	Dipole (Sumatra-Java coast / East African coast)	-
	HadISST	September	Positive	Dipole (Sumatra-Java coast / East African coast)	-
LongRunMIP (control)	CCSM3	September	Positive	Dipole (Sumatra-Java coast / East African coast)	High (3)
	CCSM3II	September	Positive	Dipole (Sumatra-Java coast / East African coast)	High (3)
	CESM104	October	Positive	Dipole (Sumatra-Java coast / East African coast)	High (3)
	CNRMCM61	December	Negative	Dipole (Sumatra-Java coast / East African coast)	Low (1)
	GFDLCM3	September	Positive	Dipole (Sumatra-Java coast / East African coast)	High (3)
	GFDLESM2M	September	Negative	Dipole (Sumatra-Java coast / East African coast)	Medium (2)
	GISSE2R	October	Positive	Dipole (Sumatra-Java coast / Arabian Sea)	Medium (2)
	HadCM3L	October	Positive	Dipole (Sumatra-Java coast / Arabian Sea)	Medium (2)
	IPSLCM5A	October	Positive	Dipole (Sumatra-Java coast / East African coast)	High (3)
	MIROC32	September	Positive	Dipole (Sumatra-Java coast / East African coast)	High (3)
	MPIESM12	August	Negative	Dipole (Sumatra-Java coast / East African coast)	Low (1)

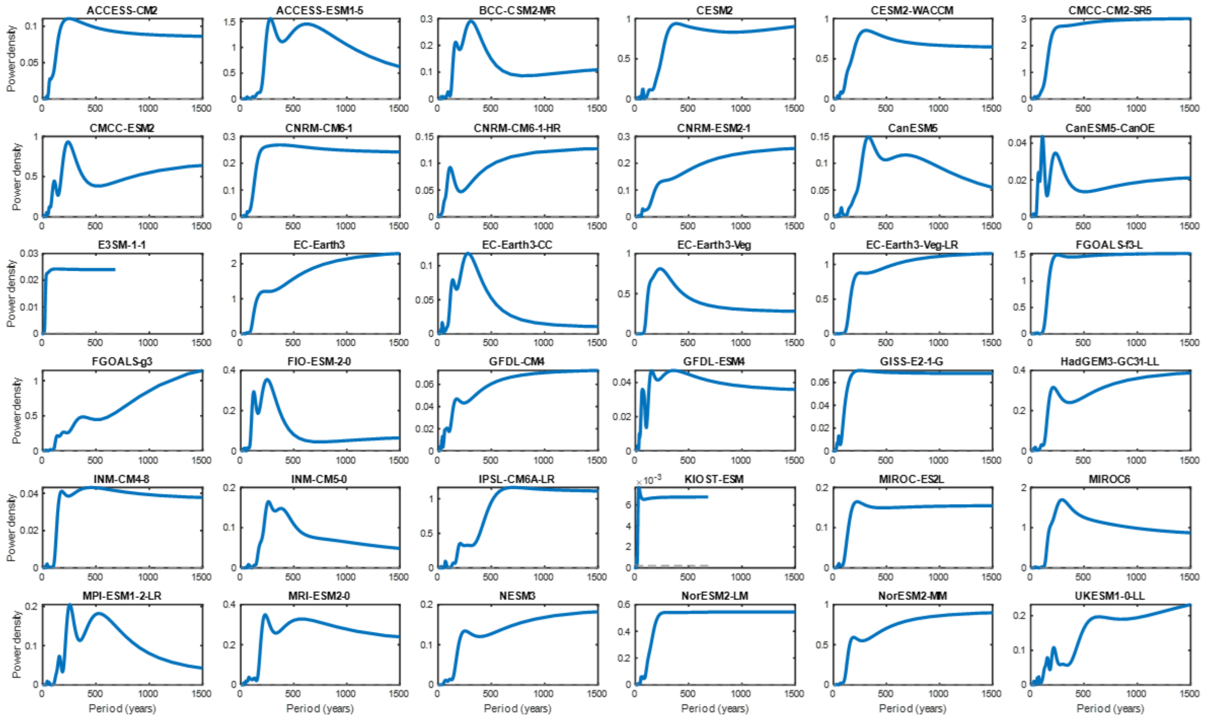

Supplementary Fig. 1. Power spectrum of the IOD amplitude in the CMIP6 piControl experiment. The power spectrum of centennial IOD amplitude changes (100-year moving standard deviation of DMI) for 36 models (Methods). The blue line is the power density. The dashed black lines are the 99 %, 95 %, and 50 % significance levels against the red noise null hypothesis (plotted in the figure, but the level is very low).

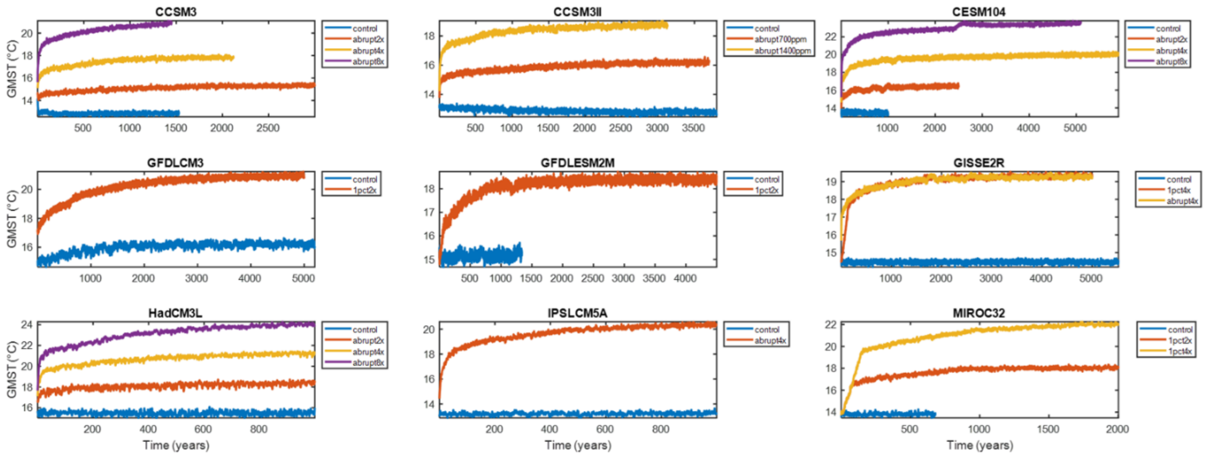

Supplementary Fig. 2. The time evolution of the GMST of the LongRunMIP simulations.

The time evolution of the annual mean GMST is shown for each model and experiment.

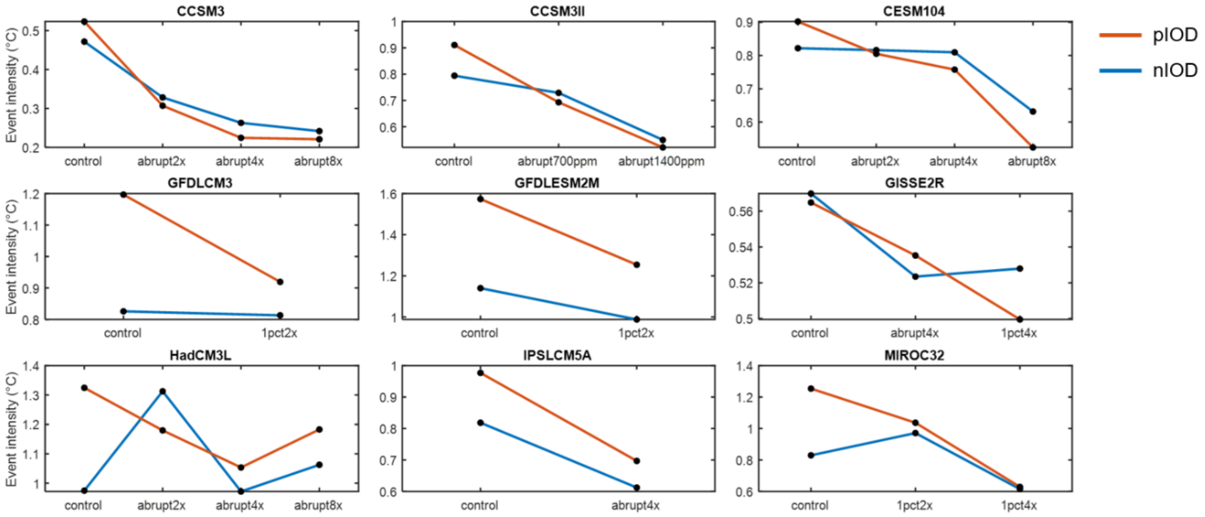

Supplementary Fig. 3. Changes in the average intensity of positive and negative IOD events. The averaged intensity of positive (orange line) and negative (blue line) IOD events during the equilibrium period for each model and experiment.

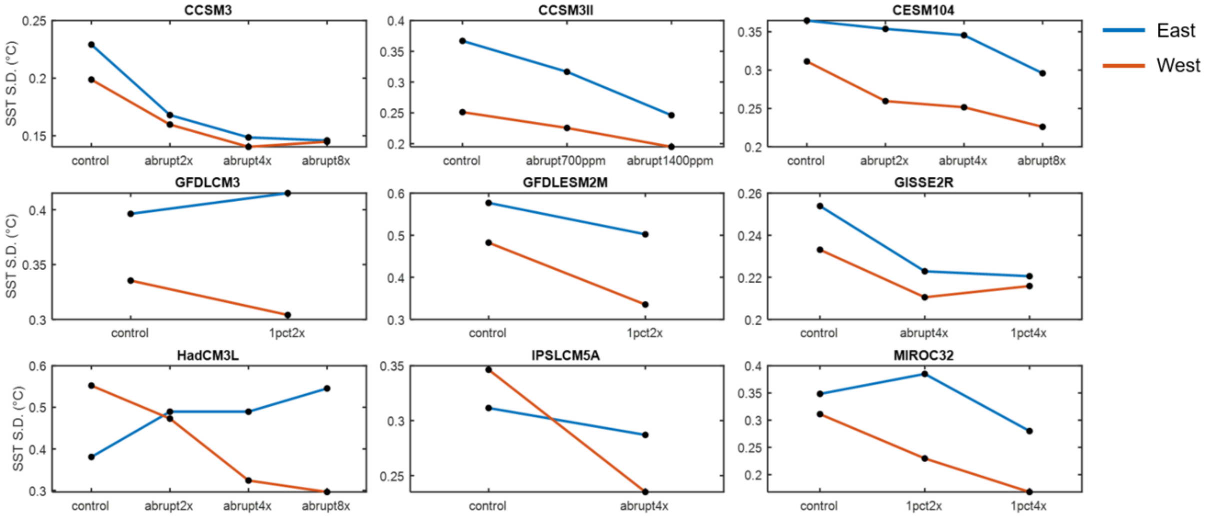

Supplementary Fig. 4. Changes in SST variability in the eastern and western tropical Indian Ocean. The standard deviation of the SST anomaly in the eastern (90° E-110° E and 10° S-0° N) (blue line) and western (50° E-70° E and 10° S-10° N) (orange line) tropical Indian Ocean during the equilibrium period.

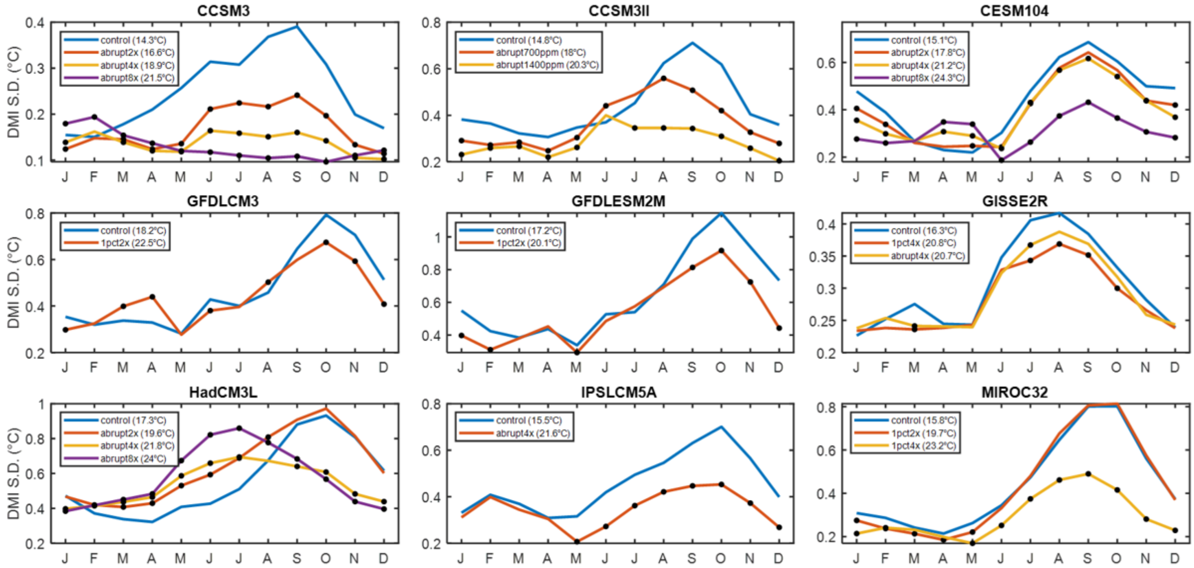

Supplementary Fig. 5. Changes in the seasonal standard deviation of DMI. The seasonal standard deviation of the DMI during the equilibrium period. The GMST of each experiment is indicated in the legend. The statistical significance of the change in the seasonal DMI standard deviation in the high-CO_2 simulation compared to the control simulation is tested with the *F*-test. The *F*-test is performed for a pair of DMI time series of the control and high-CO_2 simulations for each calendar month. The statistically significant ($P<0.05$) point is marked as a black circle.

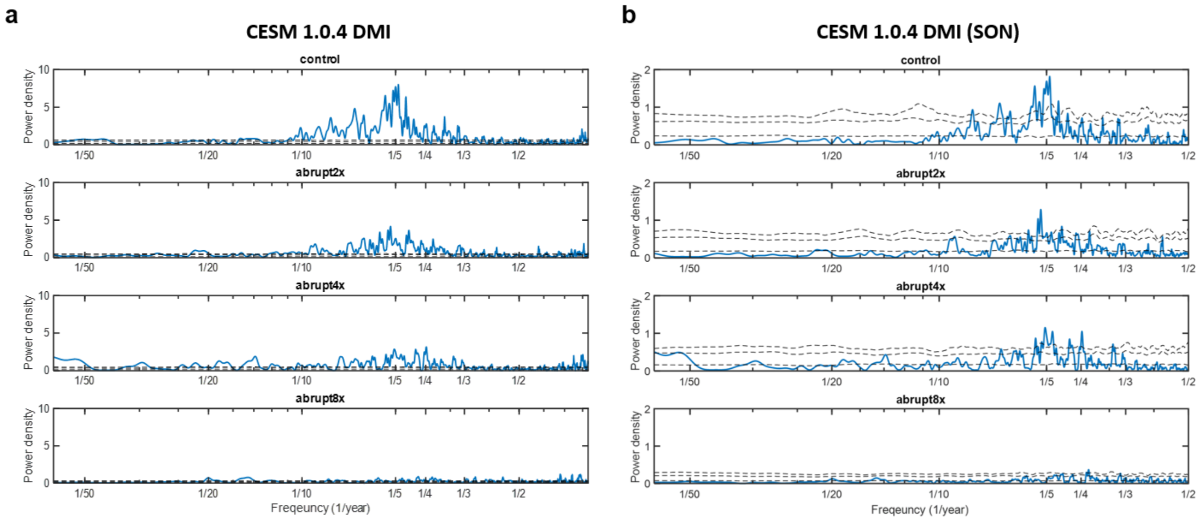

Supplementary Fig. 6. Power spectrum of the CESM1.0.4 DMI. Power spectrum of the control, abrupt2x, abrup4x, and abrupt8x of the monthly DMI (**a**) and the September-October-November averaged yearly DMI (**b**). The blue line is the power density and the dashed black lines are the 99 %, 95 %, and 50 % significance levels against the red noise null hypothesis.

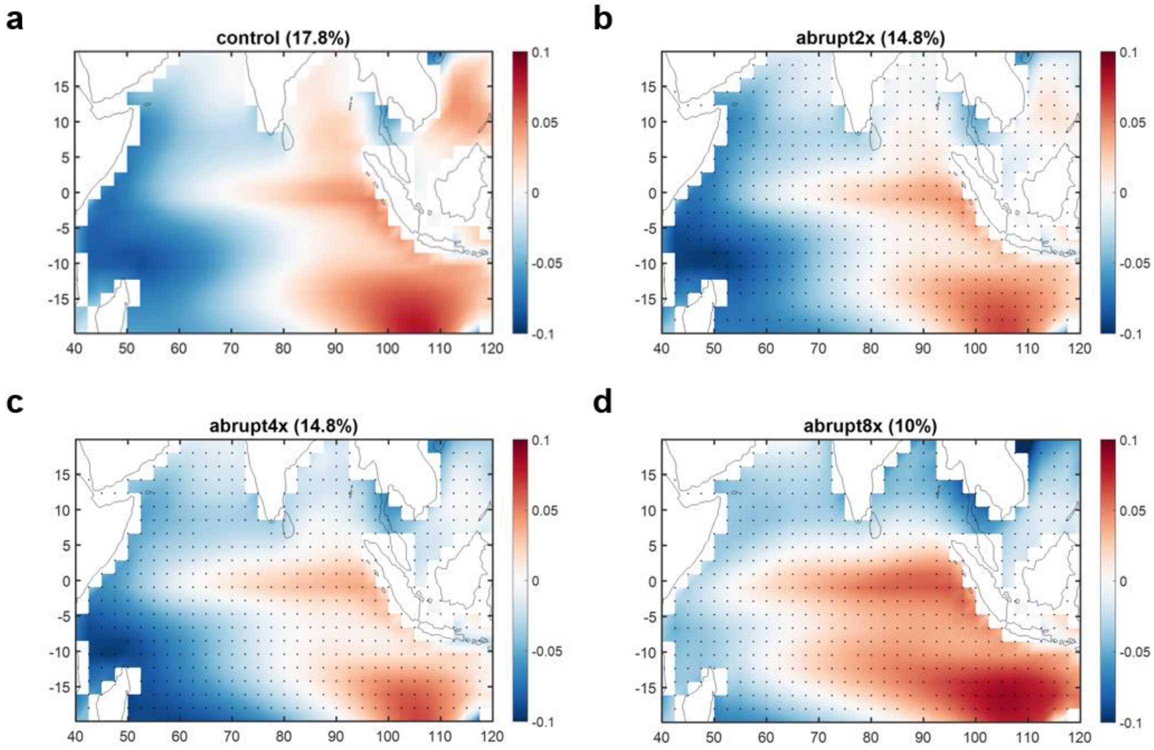

Supplementary Fig. 7. Empirical Orthogonal Function (EOF) patterns of the CESM1.0.4 surface temperature anomaly. The second EOF modes of the monthly surface temperature anomaly over the tropical Indian Ocean (40° E-120° E and 20° S-20° N). The results for the control (**a**), abrupt2x (**b**), abrupt4x (**c**), and abrupt8x (**d**). Variance explained by the first EOF mode is marked in the title of each panel. The statistical significance of the change in the second EOF mode surface temperature anomaly in the high-CO_2 simulation compared to the control simulation is tested with the *F*-test. The *F*-test is performed for a pair of the reconstructed second mode surface temperature anomalies of the control and high-CO_2 simulations for each grid point. The statistically significant ($P<0.05$) point is marked as a black circle. We note that the spatial pattern of the second EOF mode of the CESM1.0.4 shows some difference from the typical observation. Both the CESM1.0.4 and the historical observational records show a strong SST EOF pattern in the off-Sumatra region, but the CESM1.0.4 also shows a strong SST in the Sumatra coastal region. The difference can be partly attributed to the bias of the CESM1.0.4 in simulating the mean climatological state of the tropical Indian Ocean, including the wind stress

field, the SST pattern, and the ocean potential temperature profile. A detailed discussion of this subject is beyond the scope of this study which focuses primarily on the response of the tropical Indian Ocean to greenhouse gas warming. Despite such bias in the mean state, our conclusion in this paper is well supported by the strong inter-model consensus in the IOD response.

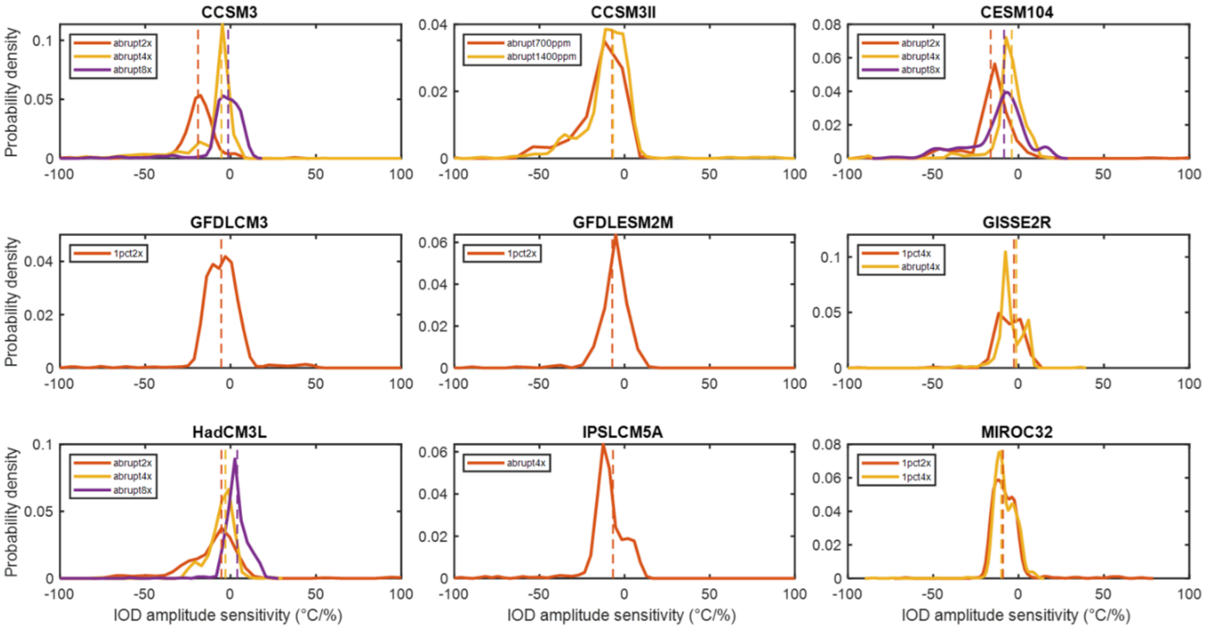

Supplementary Fig. 8. Transient sensitivity of IOD amplitude to GMST. The kernel-smoothed distribution of the transient sensitivity of IOD amplitude to GMST (solid line) (Methods). The long-term sensitivity (i.e., the slope of the linear line in Fig. 2a) is shown as a dashed line.

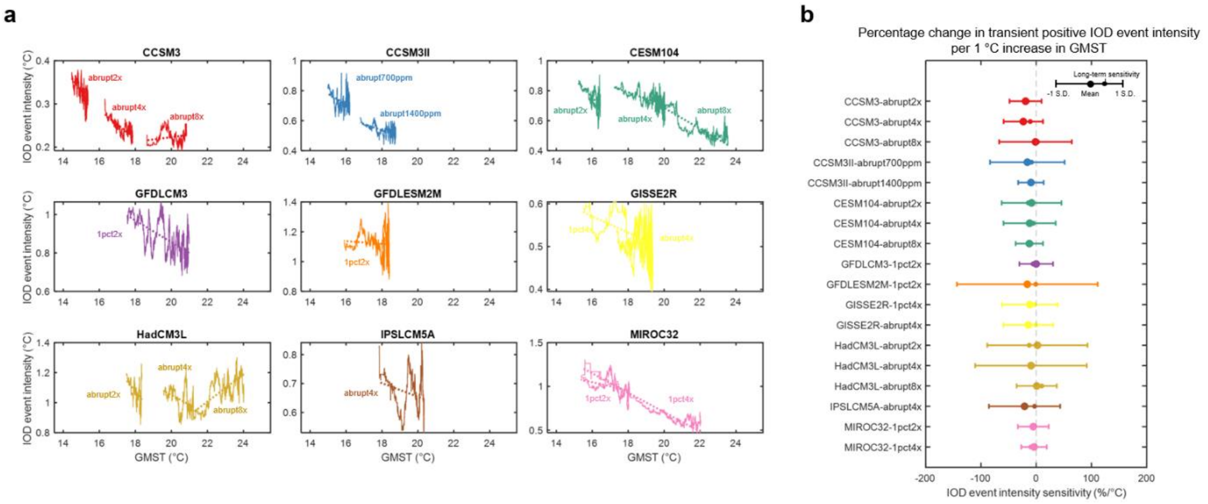

Supplementary Fig. 9. Evolution of the positive IOD intensity under greenhouse gas warming. Same as Fig. 2, but for the positive IOD event intensity. **a.** Evolution of the 100-year moving average positive IOD event intensity. The linear regression line of IOD intensity against GMST is shown as a dashed line. 15 out of 18 high-CO2 simulations show a decreasing trend in positive IOD intensity with increasing GMST. All linear trends are statistically significant (*P*<0.05) except for GISS-E2-R abrupt4x. GISS-E2-R abrupt4x shows a negative linear trend, but is not statistically significant (*P*=0.06). When counting the number of high-CO_2 simulations with a decreasing trend, GISS-E2-R abrupt4x is excluded. **b.** The statistics of the transient sensitivity of the average positive IOD event intensity. For each high-CO2 simulation, the transient sensitivity is calculated with moving GMST window of 1 °C (Methods). The mean and one standard deviation of the transient sensitivity distribution is shown as a large black circle and error bar. The long-term sensitivity (i.e., the slope of the linear regression line in a) is plotted as a small black circle.

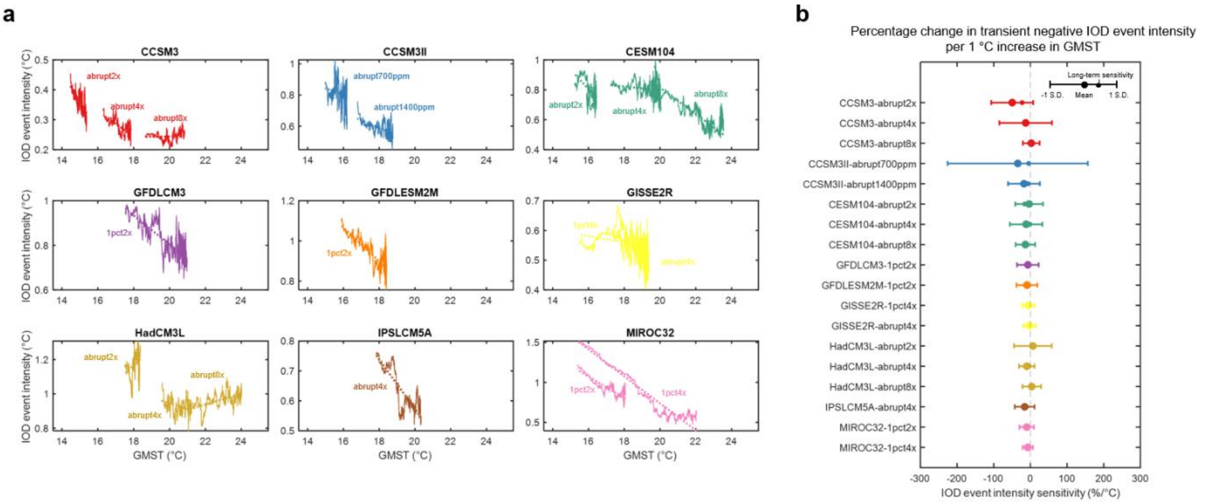

Supplementary Fig. 10. Evolution of the negative IOD intensity under greenhouse gas warming. Same as Fig. 2, but for the negative IOD event intensity. **a.** Evolution of the 100-year moving average negative IOD event intensity. The linear regression line of IOD intensity against GMST is shown as a dashed line. 15 out of 18 high-CO2 simulations show a decreasing trend in negative IOD intensity with increasing GMST. All linear trends are statistically significant (*P*<0.05) except for CCSM3 abrupt8x. CCSM3 abrupt8x shows a positive linear trend, but is not statistically significant (*P*=0.06). **b.** The statistics of the transient sensitivity of the average negative IOD event intensity. For each high-CO2 simulation, the transient sensitivity is calculated with moving GMST window of 1 °C (Methods). The mean and one standard deviation of the transient sensitivity distribution is shown as a large black circle and error bar. The long-term sensitivity (i.e., the slope of the linear regression line in a) is plotted as a small black circle.

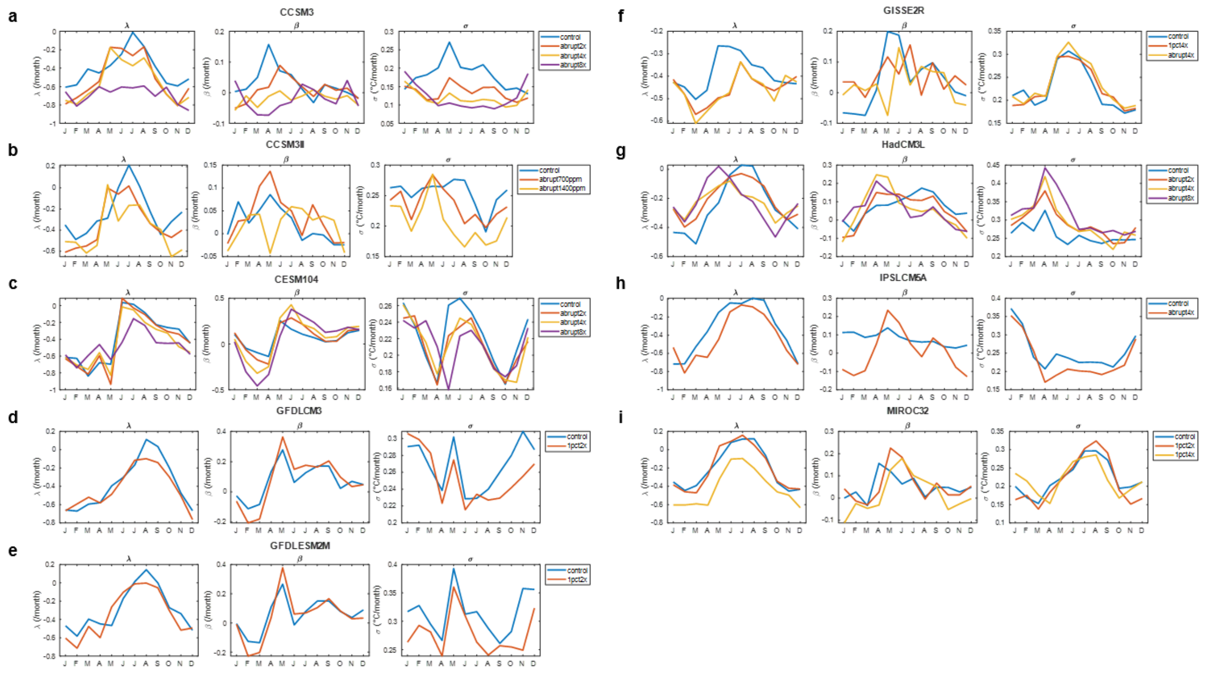

Supplementary Fig. 11. Parameters of the simple IOD model for the equilibrium period.

For each simulation, the λ, β, and σ are fitted for each calendar month.

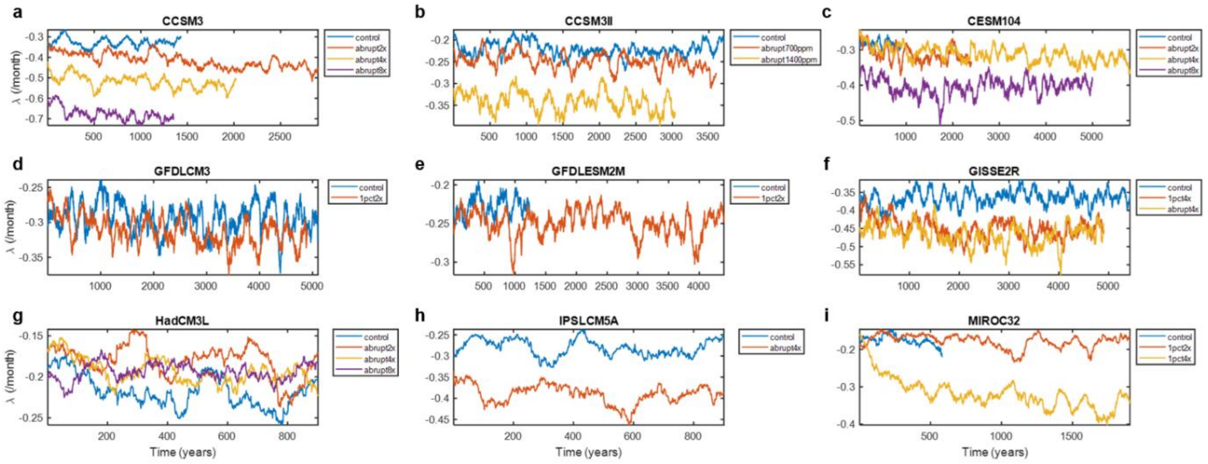

Supplementary Fig. 12. Time evolution of *λ*. For each simulation, a 100-year moving *λ* is estimated. The *λ* is estimated for each calendar month, but its annual mean value is shown for simplicity.

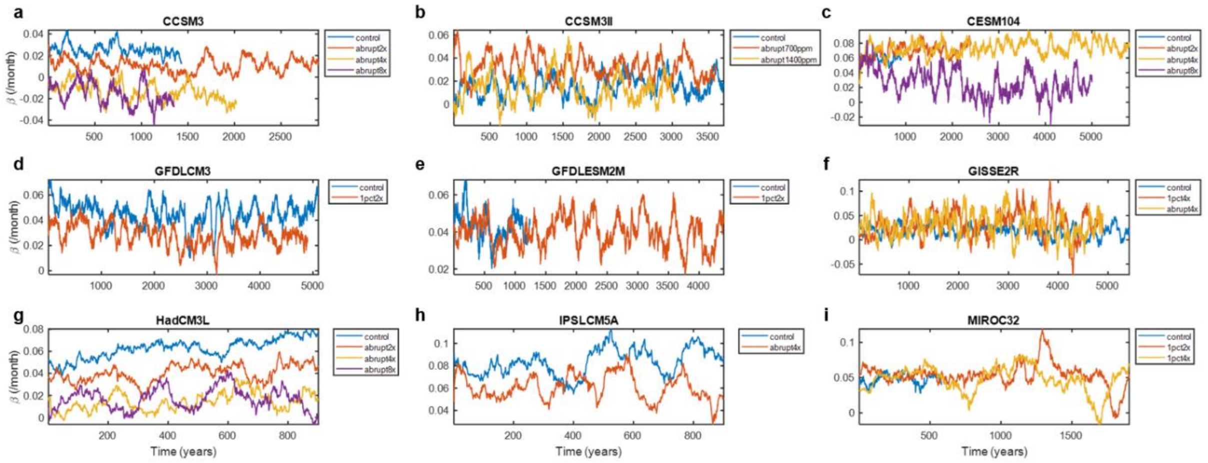

Supplementary Fig. 13. Time evolution of *β*. Same as Supplementary Fig. 12, but for *β*.

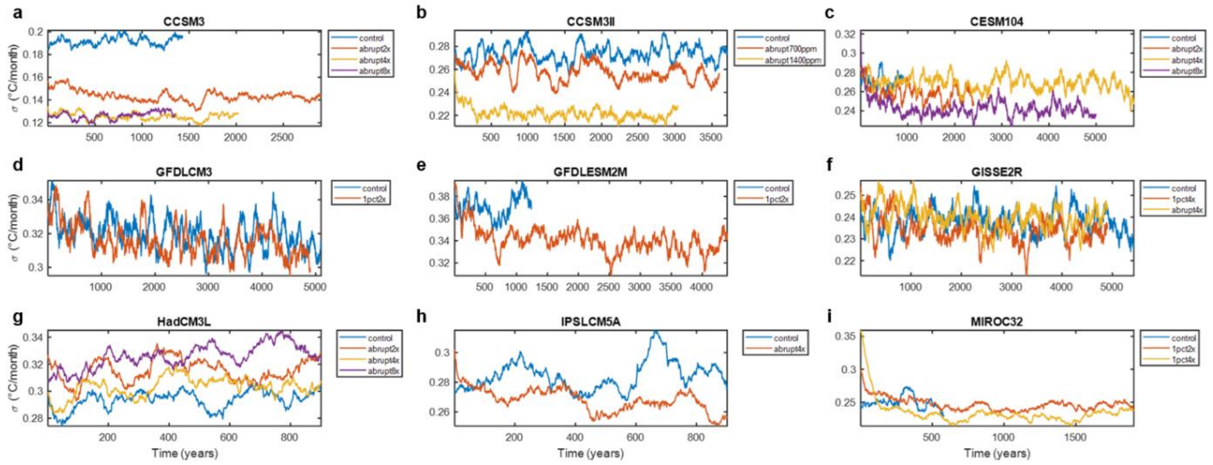

Supplementary Fig. 14. Time evolution of *σ*. Same as Supplementary Fig. 12, but for *σ*.

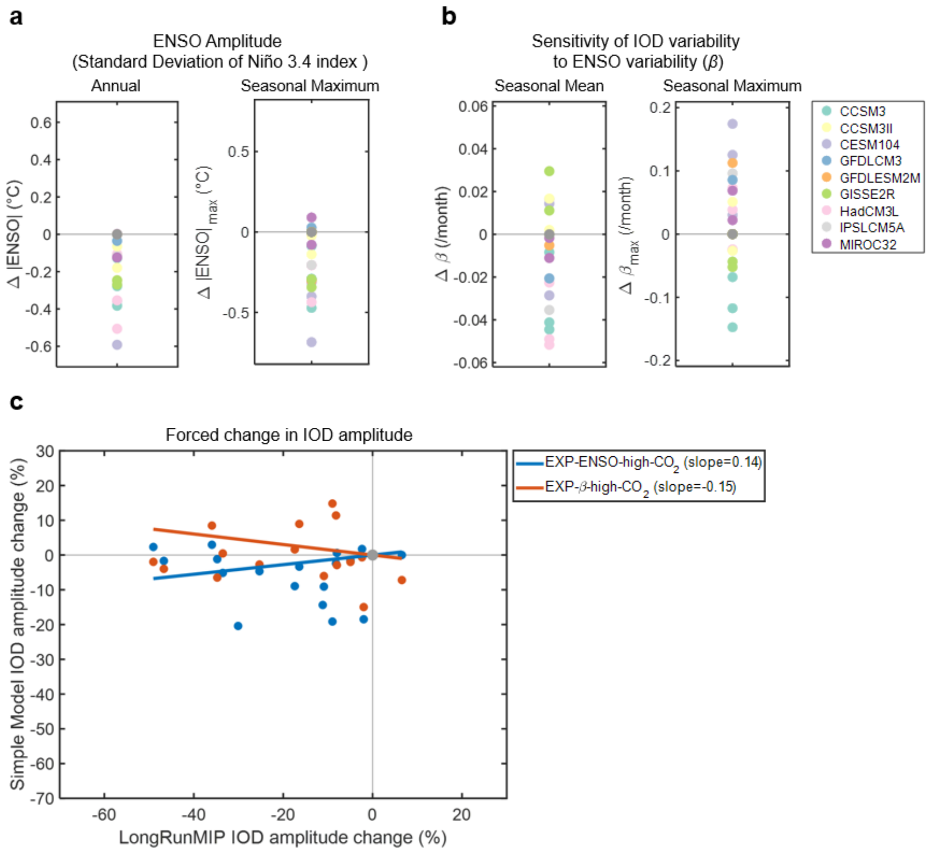

Supplementary Fig. 15. (a-b). Same as Fig. 3a, but for the ENSO amplitude and β. **a.** Change in the standard deviation of Niño 3.4 index in the equilibrium period of high-CO_2 simulation. The change is calculated by subtracting the control simulation value from the high-CO_2 simulation value (colored dots). The changes in annual standard deviation of Niño 3.4 index (left panel) and seasonally maximum standard deviation of Niño 3.4 index (right panel). **b.** Same as, **a** but for β. Seasonal mean of β (left panel) and seasonal maximum of β (right panel). **c.** Same as Fig. 3f, but for the results of the additional parameter perturbation for *ENSO* and β. The original (x-axis) and reproduced IOD amplitude by the simple IOD model (y-axis). The percentage change in IOD amplitude from the control experiment is shown. The colored dots are the results of each high-CO2 simulation. The linear regression line (without intercept) of the reproduced IOD amplitude versus the original IOD amplitude (solid line). The slope of the regression line is shown in the legend.

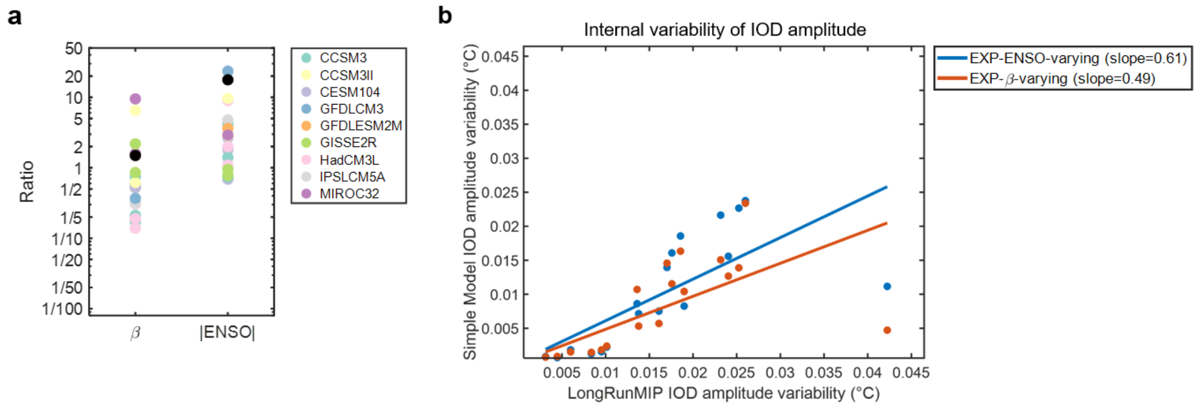

Supplementary Fig. 16. a. Same as Fig. 3e, but for the ENSO amplitude and β**.** The ratio of internal variability to forced changes. The standard deviation of the anomaly (deviation from a quadratic trend) is divided by the forced equilibrium change (deviation from the control experiment). A larger ratio indicates greater internal variability compared to the forced response. The black dot indicates the ensemble mean. **b.** Same as Fig. 3g, but for the results of the additional low-pass parameter experiment for *ENSO* and β.

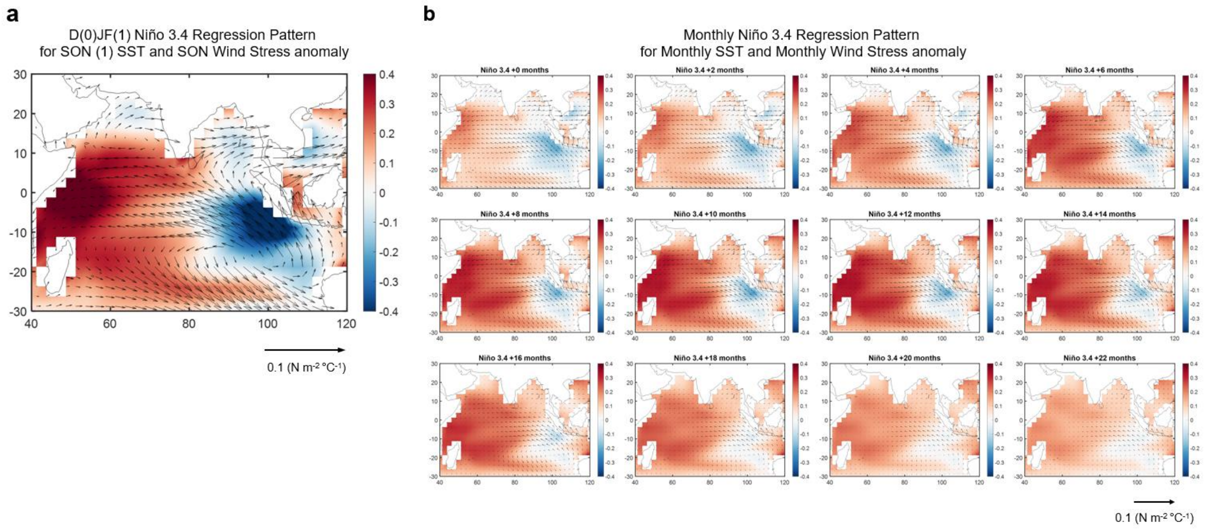

Supplementary Fig. 17. Niño 3.4 regression pattern for SST and wind stress anomaly in the tropical Indian Ocean in MIROC3.2 control simulation. a. December-January-February (D0JF1) averaged Niño 3.4 regression pattern for the following year's September-October-November (SON1) averaged SST anomaly (color shading and unitless) and wind stress anomaly (black arrow and unit is $Nm^{-2}°C^{-1}$). **b.** Lagged monthly Niño 3.4 regression pattern for monthly SST anomaly (color shading and unitless) and wind stress anomaly (black arrow and unit is $Nm^{-2}°C^{-1}$). The series of panels shows the lagged regression of the SST and wind stress anomaly against Niño 3.4 for a lag of 0 months to 22 months.

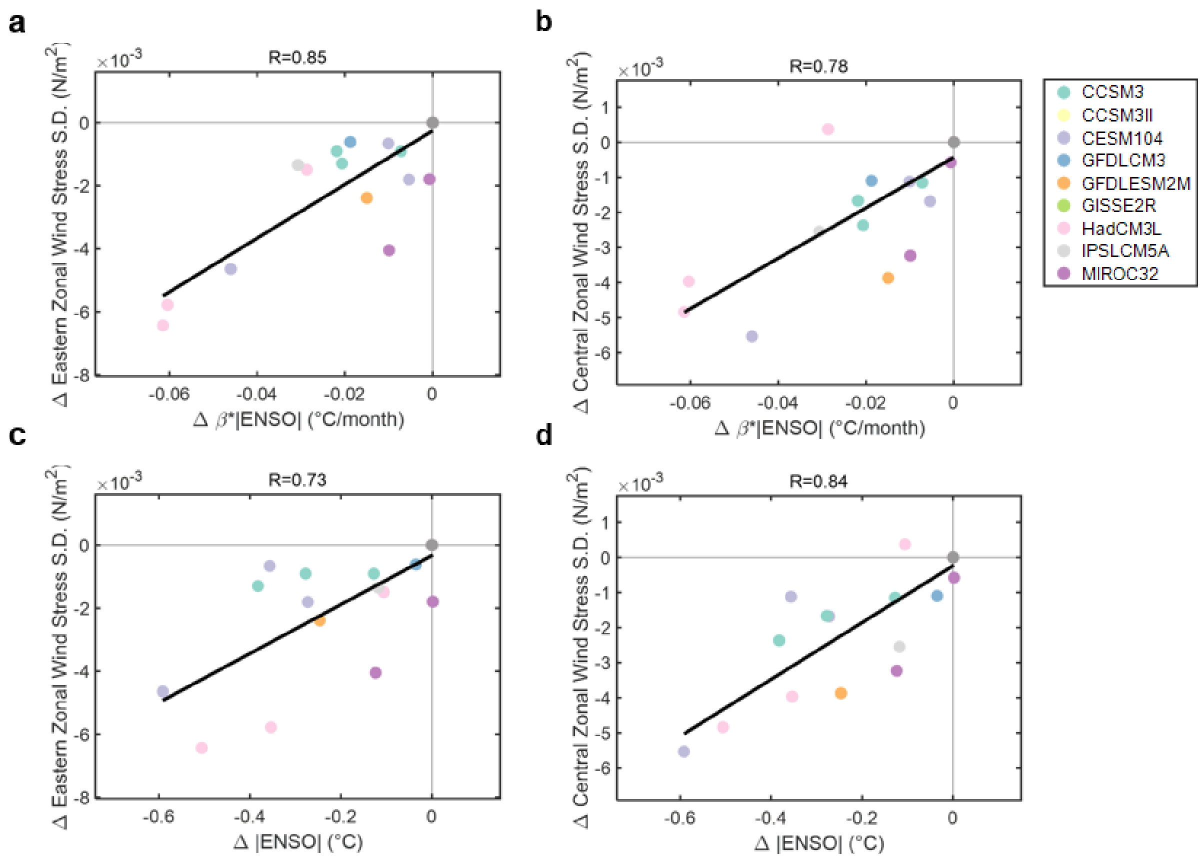

Supplementary Fig. 18. Statistical relationship between the forced changes in ENSO and strength of wind stress variability in the tropical Indian Ocean. (a-b). Change in $\beta \times ENSO$ and the standard deviation of zonal wind stress anomaly (i.e., strength of the wind stress variability). **a.** Eastern zonal wind stress anomaly (90° E-110° E and 10° S-0° N). **b.** Central zonal wind stress anomaly (70° E-90° E and 5° S S-5° N). **(c-d).** Same as (a-b), but with the standard deviation of Niño 3.4 index (i.e., ENSO amplitude). The change is calculated by subtracting the control simulation value from the high-CO_2 simulation value (colored dots). The linear regression line is shown as a black line. The Pearson correlation coefficient is indicated on the left low side of the panel. Each colored dot represents the results for each high-CO2 simulation.

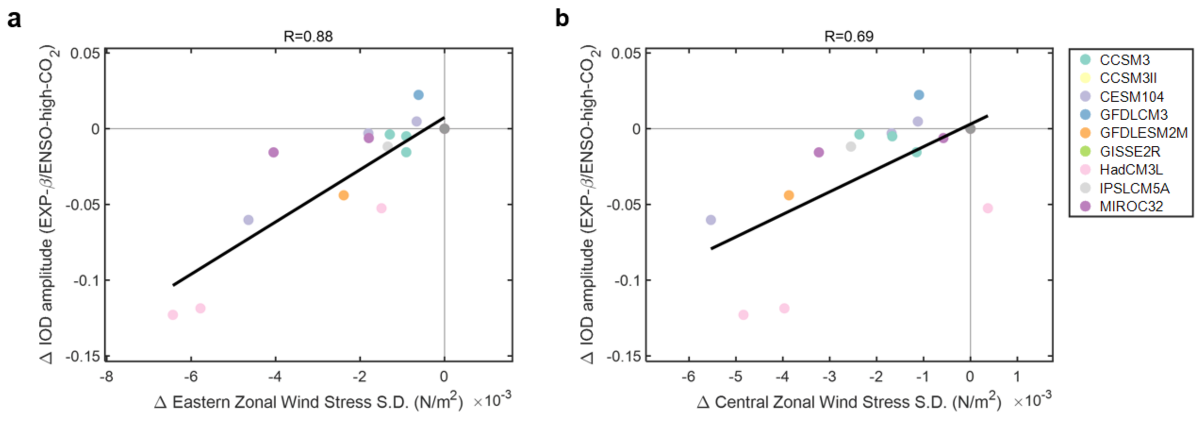

Supplementary Fig. 19. Statistical relationship between the forced change in strength of wind stress variability in the tropical Indian Ocean and ENSO-induced IOD amplitude change. Change in the standard deviation of zonal wind stress anomaly (i.e., strength of the wind stress variability) and ENSO-induced IOD amplitude change simulated by the simple IOD model experiment (EXP-β/ENSO-high-CO_2, also shown in Fig. 3f). **a.** Eastern zonal wind stress anomaly (90° E-110° E and 10° S-0° N). **b.** Central zonal wind stress anomaly (70° E-90° E and 5° S S-5° N). The change is calculated by subtracting the control simulation value from the high-CO2 simulation value (colored dots). The linear regression line is shown as a black line. The Pearson correlation coefficient is indicated on the left low side of the panel. Each colored dot represents the results for each high-CO2 simulation.

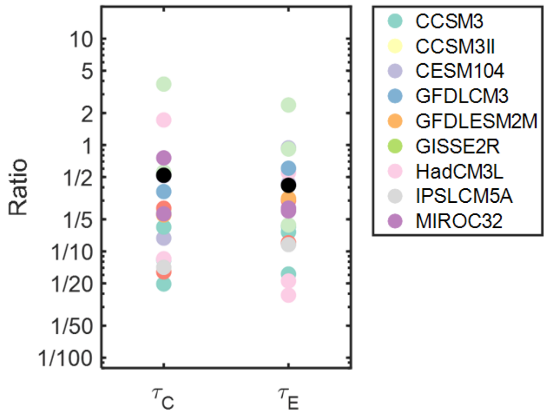

Supplementary Fig. 20. Same as Fig. 3e, but for the strength of the wind stress variability in the central (70° E-90° E and 5° S S-5° N) and eastern (90° E-110° E and 10° S-0° N) tropical Indian Ocean. The strength of the variability is measured as the standard deviation of the wind stress anomaly.

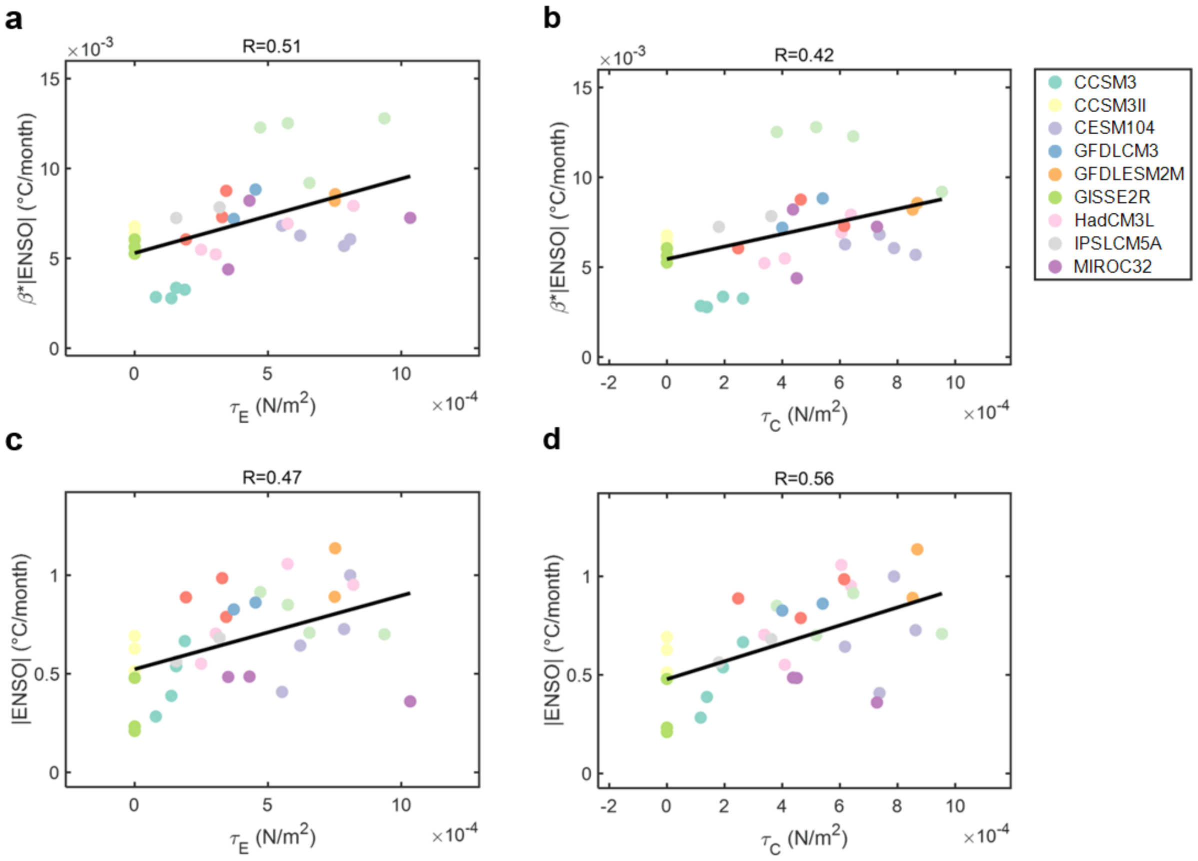

Supplementary Fig. 21. Statistical relationship between the strength of internal variability of wind stress and ENSO. (a-b). The strength of internal variability of the $\beta \times ENSO$ and wind stress anomaly. **a.** Eastern zonal wind stress anomaly (90° E-110° E and 10° S-0° N). **b.** Central zonal wind stress anomaly (70° E-90° E and 5° S S-5° N). The strength of internal variability of $\beta \times ENSO$ is measured as the standard deviation of the 100-year moving $\beta \times ENSO$. The strength of internal variability of wind stress is measured as standard deviation of 100-year moving standard deviation of wind stress anomaly. **(c-d).** Same as (a-b), but with the standard deviation of Niño 3.4 index (i.e., ENSO amplitude). The linear regression line is shown as a black line. The Pearson correlation coefficient is indicated on the left low side of the panel. Each colored dot represents the results for each LongRunMIP simulation.

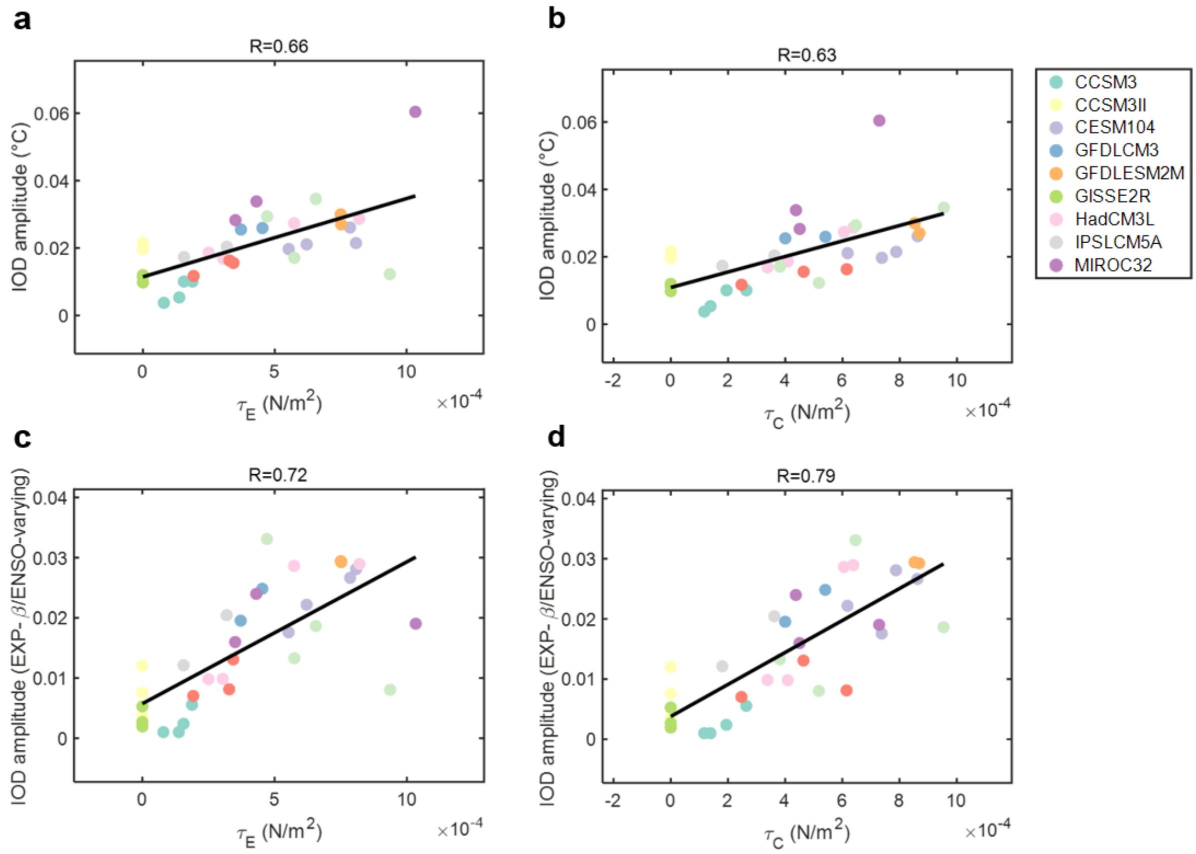

Supplementary Fig. 22. Same as Supplementary Fig. 21, but the IOD amplitude is compared with the wind stress. **(a-b).** The strength of internal variability of the IOD amplitude and wind stress anomaly. **a.** Eastern zonal wind stress anomaly (90° E-110° E and 10° S-0° N). **b.** Central zonal wind stress anomaly (70° E-90° E and 5° S S-5° N). The strength of internal variability of IOD amplitude is measured as the standard deviation of the 100-year moving IOD amplitude. The strength of internal variability of wind stress is measured as standard deviation of 100-year moving standard deviation of wind stress anomaly. **(c-d).** Same as (a-b), but with the ENSO forcing induced IOD amplitude internal variability simulated by the simple IOD model experiment (EXP-β/ENSO-varying, also shown in Fig. 3g). The linear regression line is shown as a black line. The Pearson correlation coefficient is indicated on the left low side of the panel. Each colored dot represents the results for each LongRunMIP simulation.

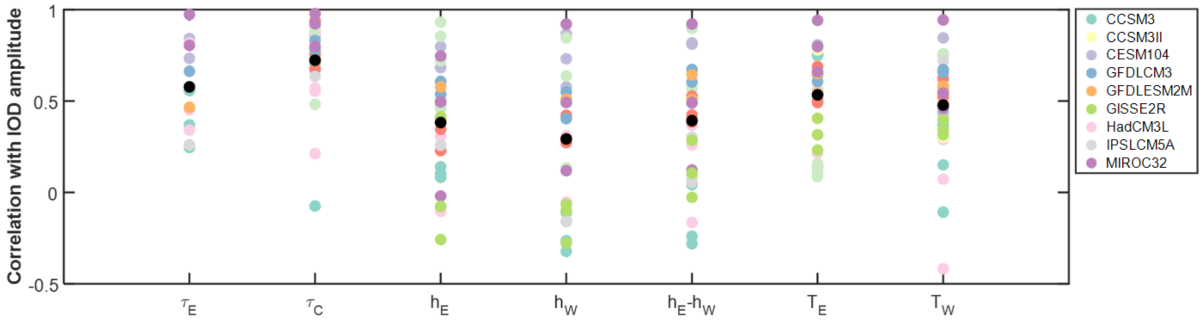

Supplementary Fig. 23. Temporal correlation between internal variability of IOD amplitude and internal variability of tropical Indian Ocean state. The correlation between 100-year moving IOD amplitude and 100-year moving standard deviation of eastern zonal wind stress anomaly (90° E-110° E and 10° S-0° N), central zonal wind stress anomaly (70° E-90° E and 5° S S-5° N), eastern thermocline depth anomaly (90° E-110° E and 10° S-0° N), western thermocline depth anomaly (50° E-70° E and 10° S-10° N), eastern minus western thermocline depth anomaly, eastern SST anomaly (90° E-110° E and 10° S-0° N), western SST anomaly (50° E-70° E and 10° S-10° N). The black dot indicates the ensemble mean.

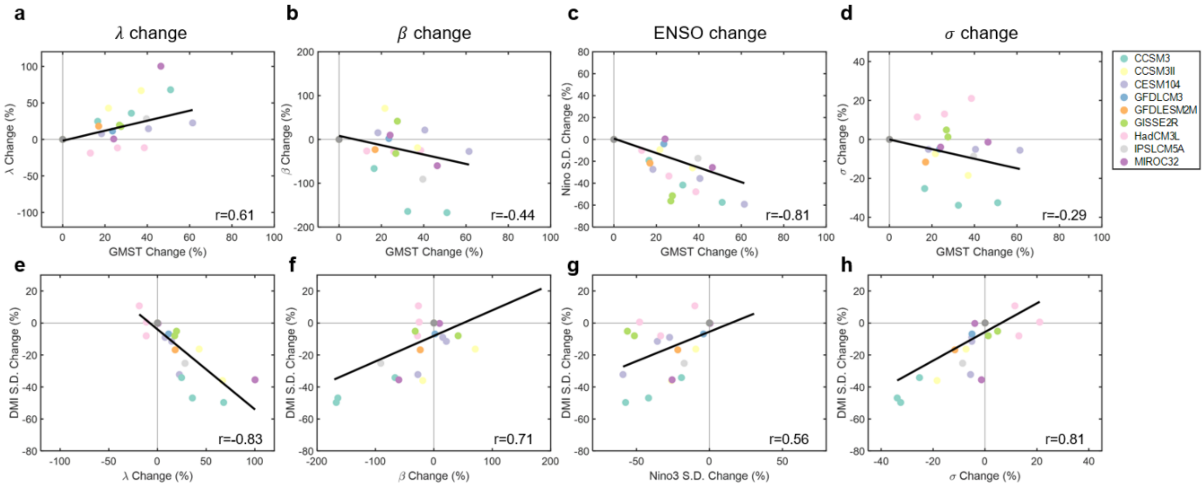

Supplementary Fig. 24. Statistical relationship between the GMST, simple IOD model parameters, and IOD amplitude changes. a-d. Relationship between changes in λ (a), β (b), standard deviation of the Niño 3.4 index (c), and σ (d) changes and GMST changes. The change is calculated as the percentage change from the high-CO_2 simulation value to the control simulation value. The linear regression line is shown as a black line. The Pearson correlation coefficient is indicated on the left low side of the panel. Each colored dot represents the results for each high-CO_2 simulation. **e-h**. Same as (a-d) but for the relationship between IOD amplitude change. The GMST change by CO2 forcing would cause the change in the model parameters and ENSO, contributing to the IOD amplitude change.

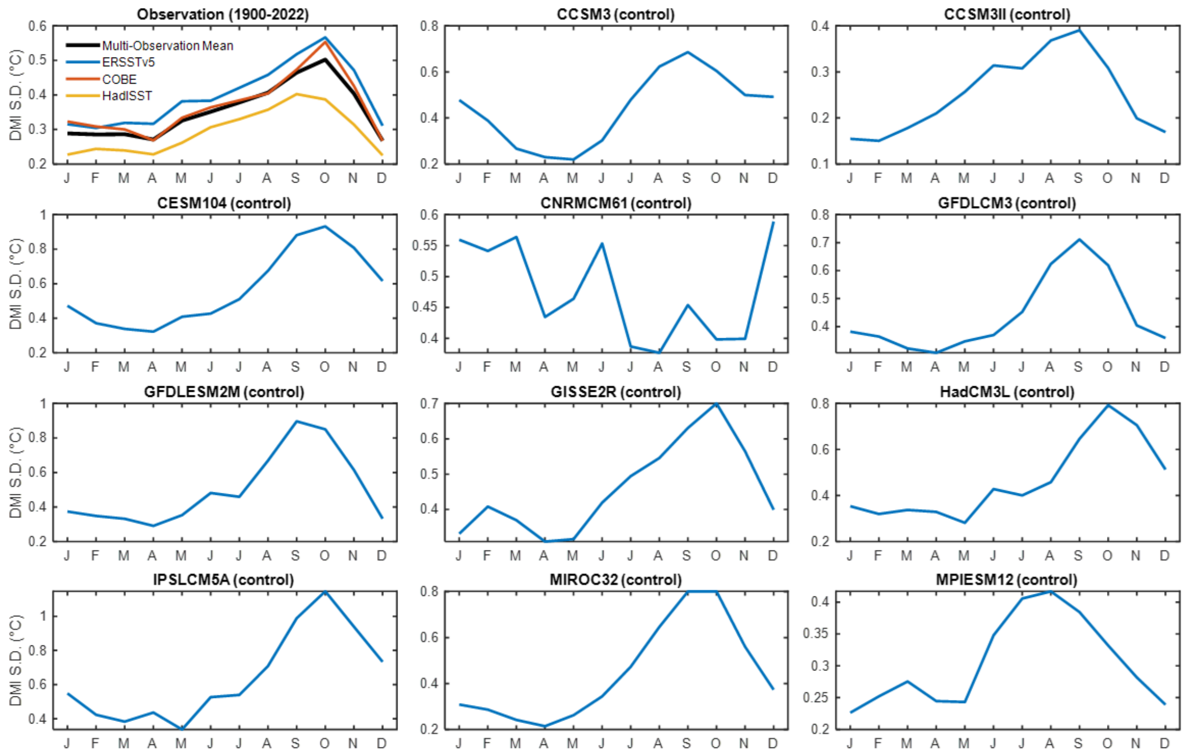

Supplementary Fig. 25. The LongRunMIP model-observation comparison for the seasonal DMI standard deviation. Note that the CNRM-CM6-1 and MPIESM-1.2 are not included in the LongRunMIP analysis sample, as they are evaluated as the low performance model (Supplementary Discussion 1 and Supplementary Table 3).

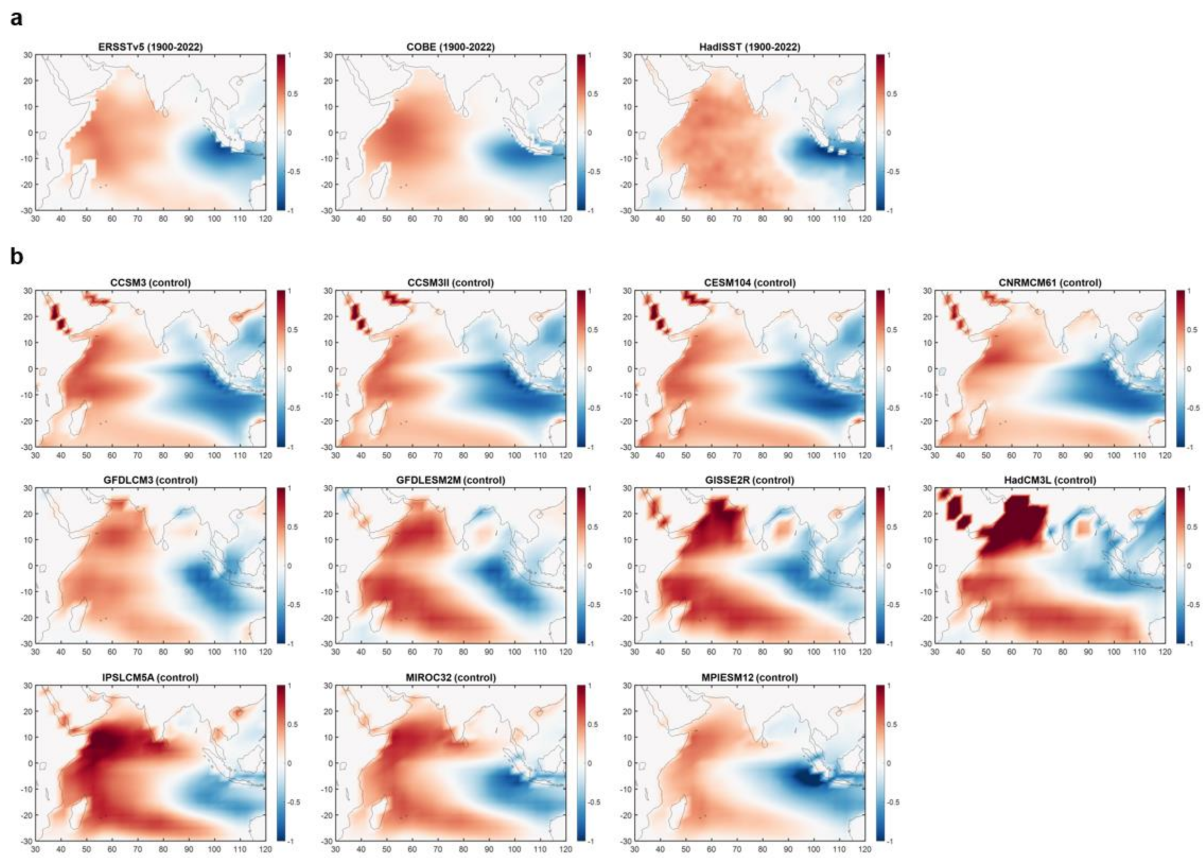

Supplementary Fig. 26. The LongRunMIP model-observation comparison for the DMI regression pattern. The DMI is regressed into the SST anomaly field for the observation (**a**) and LongRunMIP models (**b**). Note that the CNRM-CM6-1 and MPIESM-1.2 are not included in the LongRunMIP analysis sample, as they are evaluated as the low performance model (Supplementary Discussion 1 and Supplementary Table 3).

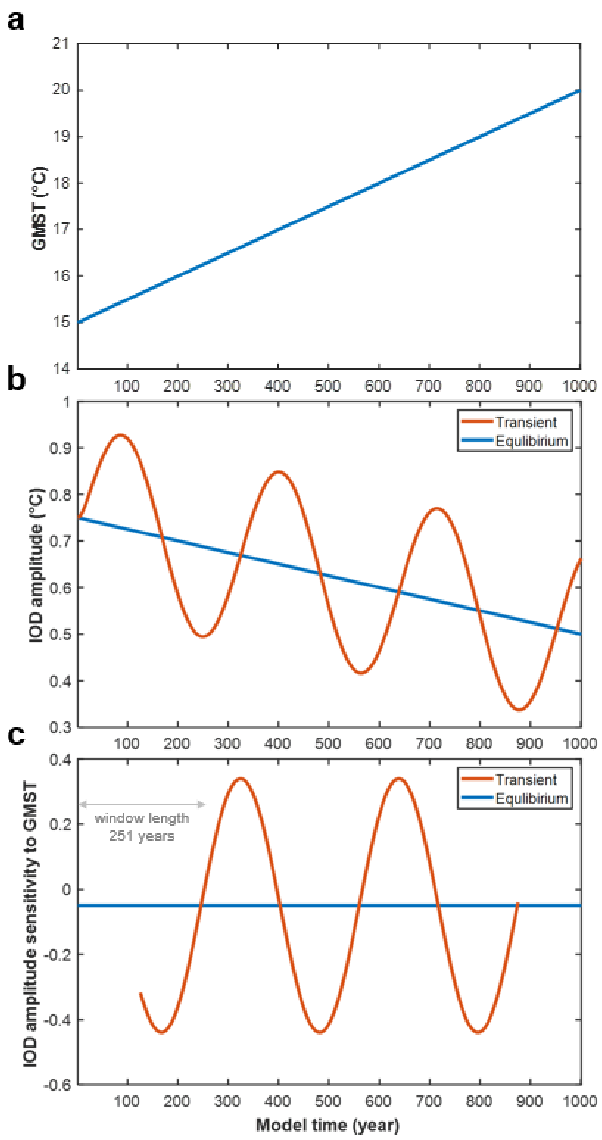

Supplementary Fig. 27. The solution for the idealized internal variability masking model. **a.** The increasing GMST forcing. **b.** The solved transient IOD amplitude (x) (orange line) and the equilibrium IOD amplitude (x_{eq}) (blue line). **c.** The 251-year moving window sensitivity of the IOD amplitude to GMST (α_{ts}) (orange line). The equilibrium sensitivity of IOD amplitude to GMST (α_{eq}) (blue line).

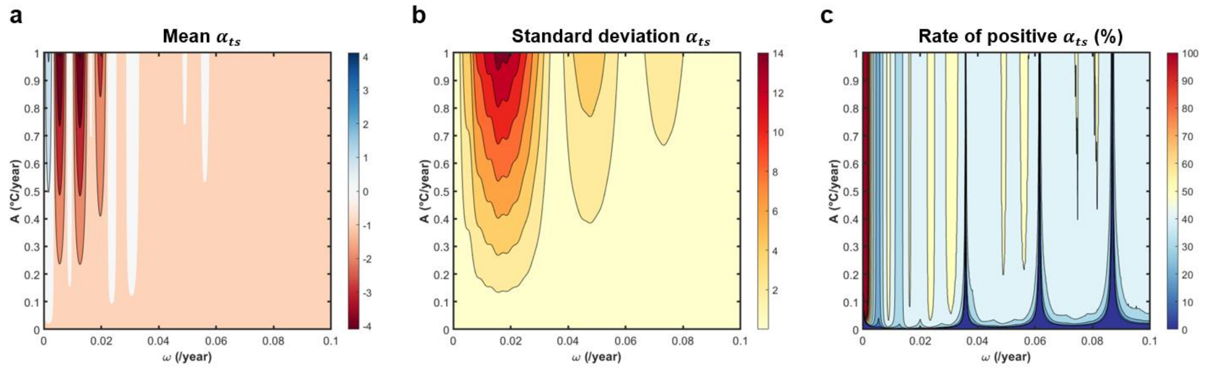

Supplementary Fig. 28. The result of the sensitivity experiment using the idealized internal variability masking model. The statistics of α_{ts} distribution. The mean (**a**) and standard deviation (**b**) of α_{ts} The percentage ratio of positive α_{ts} (**c**).

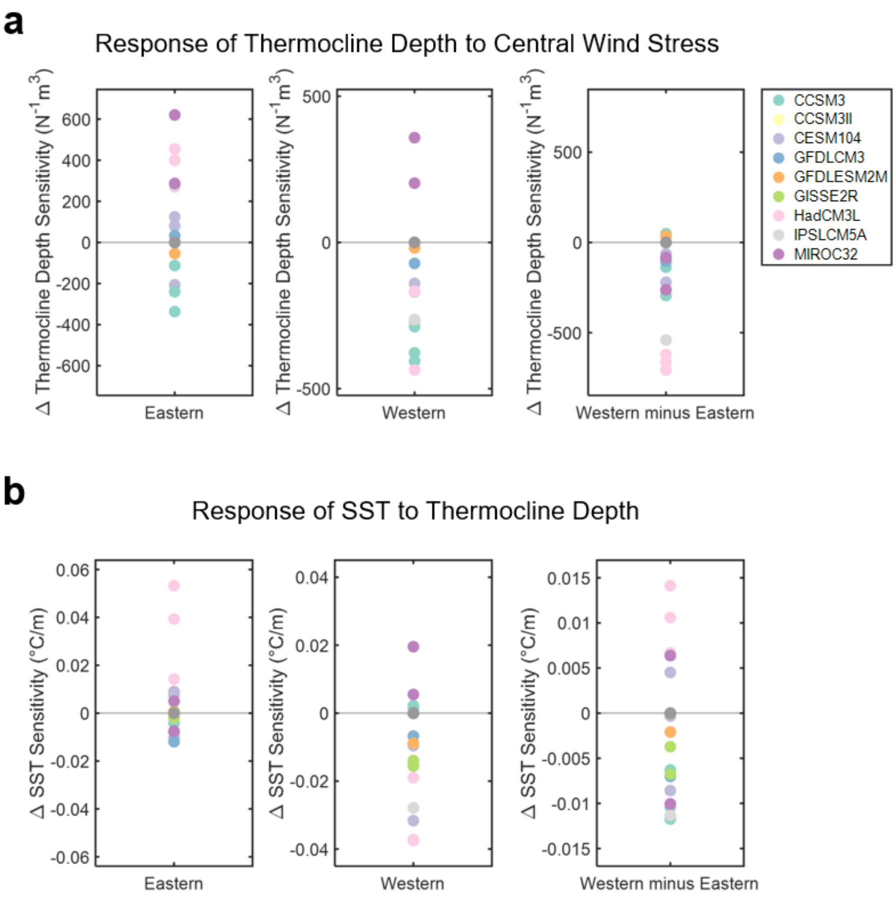

Supplementary Fig. 29. Changes in the sensitivities. a. Changes in the sensitivity of thermocline depths to the central wind stress anomaly. The results for the east (90° E-110° E and 10° S-0° N) (left panel) and west (50° E-70° E and 10° S-10° N) (middle panel) the tropical Indian Ocean thermocline depth anomaly. The west minus east thermocline depth gradient (right panel). The change is calculated as the high-CO_2 simulation minus its control simulation value. Each colored dot is the result for each model. **b.** Same as a, but for the changes in sensitivity of SST to thermocline depth anomaly. The sensitivity of the east (west) SST anomaly to the east (west) thermocline depth anomaly is shown in the right (middle) panel. The sensitivity of the west minus east SST gradient to the west minus east thermocline depth gradient anomaly is shown in the right panel. Note that the HadCM-3L and MPI-ESM-1.2 are the exceptional models that show an increase in IOD amplitude in high-CO_2 simulations.

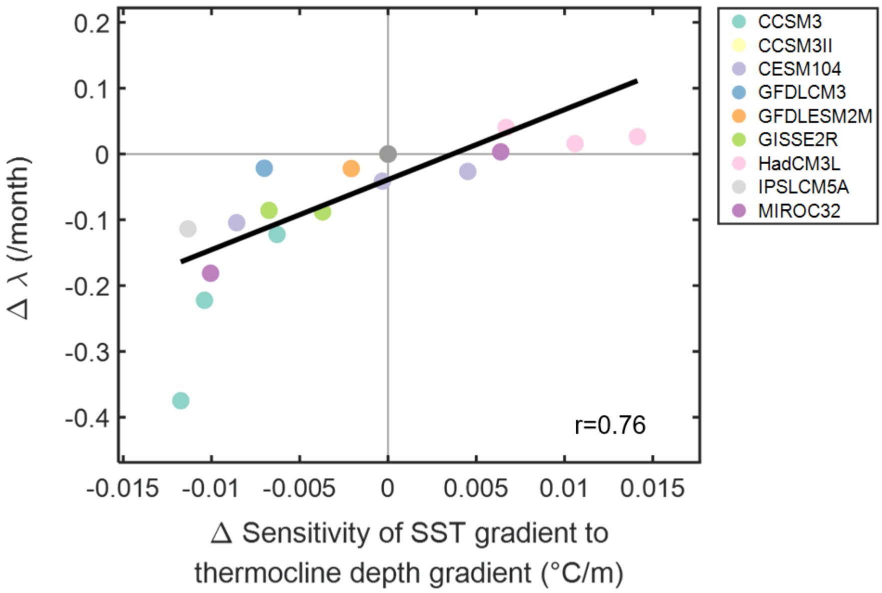

Supplementary Fig. 30. Statistical relationship between changes in λ and sensitivity of SST gradient to thermocline depth gradient. The changes in sensitivity of the SST gradient to the thermocline depth gradient shown in the right panel in Supplementary Fig. 29b are compared with the changes in λ. Each colored dot is the result for each model. The linear regression line is marked as a black line. The Pearson correlation coefficient value is 0.76.

Supplementary Discussion 1. LongRunMIP model evaluation and selection

We evaluate the IOD simulation performance of the all-available models from the LongRunMIP archive and select the models that can simulate the basic temporal and spatial characteristics of the IOD. A total of 11 LongRunMIP models are available to investigate the IOD response to greenhouse gas warming (see Supplementary Table 3). These models provide the monthly surface temperature (or monthly surface air temperature) output longer than 500 years, allowing us to reliably disentangle the internal variability and forced response of IOD to greenhouse gas warming.

We use the control simulation for the model evaluation (each model has a single number of control experiments). We specifically use the last 500 years of the control simulation to unify the temporal length of the evaluation period. For the evaluation, we use three observation products, ERSSTv5 (ref. [1]), COBE[2], and HadISST[3]. We use the period 1900-2022.

We evaluate the model performance based on the DMI, the main analysis variable of this study. We examine three key characteristics: peak DMI standard deviation season, DMI skewness sign, and the spatial DMI regression pattern. We calculate the DMI and related metrics for the LongRunMIP control simulations and the observation. The DMI calculation method is outlined in the Methods section of the main paper. Note that the LongRunMIP does not provide the historical simulation, thus the exact one-to-one quantitative comparison with the observation is not plausible (such as measuring root-mean-square error). Alternatively, we evaluate whether or not the model simulates the key observed characteristics of the IOD.

(1) Peak DMI standard deviation season. The observation shows the DMI standard deviation peak in either September or October (yet there is inter-observation diversity) (Supplementary Fig. 25). The 9 out of 11 models show a peak in either September or October. The exceptions are CNRM-CM6-1 and MPIESM-1.2, which peak in December and August,

respectively. In particular, CNRM-CM6-1 shows a very weak seasonal phase locking pattern, indicating that CNRM-CM6-1 is not able to correctly simulate the seasonal evolution of IOD.

(2) DMI skewness sign. The observation shows a positive skewness value. The 8 out of 11 models simulate positive skewness. The exceptions are CNRM-CM-61, GFDL-ESM2M, and MPIESM-1.2, which simulate negative skewness.

(3) Spatial DMI regression pattern. The observation shows a dipole pattern with peaks located on the Sumatra-Java coast and the East African coast (Supplementary Fig. 26). 9 out of 11 models show the same pattern type and peak position as the observation. The exceptions are GISS-E2-R and HadCM3L. These two models simulate a dipole pattern, but the peak is located in the Arabian Sea, not on the East African coast.

The evaluation of these metrics is summarized in Supplementary Table 3. We calculate the model performance score based on whether or not the model can simulate these three metrics. If the model simulates the given metric correctly, then the model receives a score of 1. For example, if the model simulates the peak DMI standard season in September or October, in line with the observation, then the model receives a score of 1. Each metric has an equal score of 1. The total score ranges from 0 to 3. We classified the model performance level based on the total score: high (score 3), medium (score 2), low (score 1), and very low (score 0).

Out of a total of 11 models, 6 models are rated as a high performance model, 3 models are rated as a medium performance model, and 2 models are rated as a low performance model. None of the model is rated as a very low performance model. We exclude the low performance models, and use only the high and medium performance models for this study. Finally, the following 9 models are selected: CCSM3, CCSM3II, CESM1.0.4, GFDL-CM3, GFDL-ESM2M, GISS-E2-R, HadCM3L, IPSL-CM5A, MIROC3.2. These selected models are capable of reasonably simulating the basic IOD characteristics.

Supplementary Discussion 2. Quantitative description of the internal variability masking effect

We introduce the idealized model for the internal variability masking effect to quantitatively investigate the influence of internal variability on the forced IOD amplitude change. The time evolution of IOD amplitude under greenhouse gas warming can be described as:

$$\frac{d}{dt}x = -\kappa(x - x_{eq}) + I$$

$$x_{eq} = \alpha_{eq} \times GMST + \beta_{eq}$$

$$I = Asin(\omega t)$$

The IOD amplitude (x) is adjusted towards the equilibrium amplitude (x_{eq}) at the rate of inertia parameter (κ) under the influence of the internal variability (I). The equilibrium amplitude (x_{eq}) depends linearly on the GMST ($GMST$) with equilibrium sensitivity (α_{eq}). The internal variability (I) varies sinusoidally with amplitude A and angular frequency of ω. Note that this model is different from the simple IOD model presented in the main text of the paper. The simple IOD model explicitly resolves each physical source of IOD variability (local feedback, ENSO forcing, and weather forcing) and does not explicitly account for the global warming response to IOD variability. In contrast, this idealized model collectively incorporates the response of the physical processes of IOD to global warming and encapsulates it in a single form of the equation. Such simplicity facilitates an understanding of the role of internal variability.

We first demonstrate the solution of the idealized model. We perform an idealized warming experiment. The GMST is linearly increased from 15 °C to 20 °C at a rate of

5 °C/1000 year for 1000 years (Supplementary Fig. 27a). The parameters are set as κ=0.1 (/year), α_{eq}=-0.05, β_{eq}=1.5 (°C), A=0.02 (°C/year), ω=0.02 (/year). The numerical integration is performed using the Euler method with a time interval of 0.01 years. Consistent with the LongRunMIP simulations, the IOD amplitude fluctuates with time (Supplementary Fig. 27b) and its 251-year moving transient sensitivity to GMST (α_{ts}) largely varies with time due to the internal variability (Supplementary Fig. 27c).

Next, we perform the identical idealized warming experiment, but with different values of A and ω to investigate the effect of the amplitude and frequency of the internal variability on the α_{ts}. A is varied from 0.0 (°C/year) to 1.0 (°C/year) with an interval of 0.002 (°C/year) and ω is varied from 0.0 (/year) to 0.1 (/year) with an interval of 0.0002 (/year). The statistics of the α_{ts} distribution are shown in Supplementary Fig. 28. The standard deviation of α_{ts} increases as ω decreases and A increases. For the experiments with the same value of ω, the standard deviation of α_{ts} monotonically increases as A increases. For the experiments with the same value of A, the standard deviation of α_{ts} is maximized at ω=0.02 (/year) which corresponds to the internal variability period of 314 years. Following the spread of α_{ts}, the percentage rate of positive α_{ts} in the ensemble also increases as the A increases and ω decreases. However, the detailed response pattern is complex. It implies that the measurable statistics of the IOD amplitude transient sensitivity in practice depend in a complicated way on the characteristics of the internal variability.

Supplementary Discussion 3. Relationship between the local feedback process and mean climatological state of the tropical Indian Ocean

In the equilibrium period of the high-CO_2 simulations, the local feedback process is robustly weakened (Fig. 3a). The local feedback process is associated with the mean climatological state in the tropical Indian Ocean[4–6]. Here, we discuss how the changes in the mean state of tropical Indian Ocean can contribute to weakening of the local feedback processes.

In the high-CO_2 simulations, the tropical Indian Ocean shows a robust forced change in mean climatological states; the eastern and western thermocline depths decrease (Fig. 3b), the zonal thermocline depth gradient decreases (i.e., increasing west-to-east gradient) (Fig. 3b), the zonal SST gradient decreases (Fig. 3c), and the central wind stress shows anomalous easterlies (Fig. 3d). Each of these changes contributes to either strengthening or weakening local feedback processes.

Shoaling of the thermocline depth increases the sensitivity of the thermocline depth to the zonal wind stress anomaly (Supplementary Fig. 29a). This change would intensify the wind-thermocline-SST feedback[4,5]. The increased climatological west-to-east thermocline depth gradient (Fig. 3b) due to the strengthened easterly wind would also enhance the thermocline-SST feedback[4,5] as well. The enhanced easterly wind would also enhance the wind-evaporation-SST feedback[4,5,7]. Conversely, the reduced east-to-west SST gradient (Fig. 3c) would weaken the surface zonal advective feedback[4,5]. The sensitivity of the east-to-west SST gradient to thermocline depth decreases (Supplementary Fig. 29b) and this would weaken the thermocline-SST feedback[4,5].

Note that we do not perform an inter-model correlation analysis between the mean states of the tropical Indian Ocean and the strength of the local feedback process (or IOD

amplitude) (e.g., the relationship between thermocline depth and λ), as this may potentially mislead the interpretation of the results. For example, the correlation between the changes in thermocline depth and IOD amplitude would be calculated as positive, even though the decrease in thermocline depth would physically increase the IOD amplitude by enhancing thermocline-related feedbacks. The inconsistency between the independent physical effect of the mean state variable and the superficial statistical relationship obscures the understanding of the effect of mean state changes from a physical perspective. Despite this caveat, we found that there is a high inter-model correlation between the sensitivity of the zonal SST gradient to the thermocline depth gradient and λ (r=0.77) (Supplementary Fig. 30). This shows that the sensitivity of the SST gradient to thermocline depth gradient can be a good statistical predictor of the local feedback process.

The ocean current variable is not publicly available in the LongRunMIP archive. Also, the ocean net heat flux (variable name: surf) is not consistently available throughout the model. Therefore, a quantitative analysis of the local feedback process (i.e., mixed layer budget analysis) cannot be performed using the currently available public dataset for LongRunMIP.

Supplementary References

1. Huang, B. *et al.* Extended Reconstructed Sea Surface Temperature, Version 5 (ERSSTv5): Upgrades, Validations, and Intercomparisons. *J. Clim.* **30**, 8179–8205 (2017).

2. Ishii, M., Shouji, A., Sugimoto, S. & Matsumoto, T. Objective analyses of sea-surface temperature and marine meteorological variables for the 20th century using ICOADS and the Kobe Collection. *Int. J. Climatol.* **25**, 865–879 (2005).

3. Rayner, N. A. *et al.* Global analyses of sea surface temperature, sea ice, and night marine air temperature since the late nineteenth century. *J. Geophys. Res. Atmos* **108**, 4407 (2003).

4. Cai, W. *et al.* Projected response of the Indian Ocean Dipole to greenhouse warming. *Nat. Geosci.* **6**, 999–1007 (2013).

5. Zheng, X.-T. *et al.* Indian Ocean Dipole Response to Global Warming in the CMIP5 Multimodel Ensemble. *J. Clim.* **26**, 6067–6080 (2013).

6. An, S.-I. *et al.* Intensity changes of Indian Ocean dipole mode in a carbon dioxide removal scenario. *npj Clim. Atmos. Sci.* **5**, 20 (2022).

7. An, S.-I. A dynamic link between the basin-scale and zonal modes in the Tropical Indian Ocean. *Theor. Appl. Climatol.* **78**, 203–215 (2004).
